# The Impact of Community Housing Characteristics and Epidemic Prevention Measures on Residents’ Perception of Epidemic Prevention

**DOI:** 10.3390/ijerph18147289

**Published:** 2021-07-07

**Authors:** Chi-Tz Kuo, Hsiao-Jui Sue, Po-Han Chen

**Affiliations:** 1 Department of Civil Engineering, National Taiwan University, Taipei 10617, Taiwan; pohanchen@ntu.edu.tw; 2 Department of Land Economics, National Chengchi University, Taipei 11601, Taiwan; 103257501@nccu.edu.tw

**Keywords:** congregate housing, community, COVID-19, community infection prevention, infection prevention awareness

## Abstract

Since the outbreak of COVID-19, many parts of the world have fallen into deep recession. Governments in every country have adopted various measures to restrict social gatherings due to the need to control the pandemic. This includes restrictions on activities in homes and communities. Fundamentally, epidemic prevention relies on the measures individuals take. A community’s epidemic prevention measures become more critical as activities are held in houses or communities once again. From the perspective of the theory of planned behavior, this study investigates whether the various epidemic prevention measures and characteristics of a community affect residents’ perception of epidemic prevention. We use the truncated regression model as the primary research method. The empirical results show that the community’s epidemic prevention measures can change residents’ awareness of the importance of epidemic prevention. Moreover, the scale of the community and management committee are also found to have a partial impact.

## 1. Introduction

Since the outbreak of the new coronavirus at the end of 2019, the world has been severely affected by the pandemic. According to the International Labor Organization [1], as of 25 January 2021, the loss of working hours was estimated to be equivalent to 255 million full-time jobs. In addition, total employment loss was estimated to stand at 114 million [1]. As of March 2021, more than 2.6 million people have since died from the disease [2]. According to Carmen Reinhart, Chief Economist at the World Bank Group, it will take at least five years for total output to return to pre-pandemic levels [3]. This represents the largest negative impact on the world since the global financial crisis [4].

COVID-19 was caused by a coronavirus. Therefore, its transmission mode and characteristics are similar to that of other coronaviruses [5]. According to the COVID-19 prevention guidelines published by the Centers for Disease Control and Prevention [6] in the United States, infections occur mainly through exposure to respiratory droplets when a person is in close contact with someone who has COVIO-19, and respiratory droplets cause infection when they are inhaled or deposited on mucous membranes, such as those that line the inside of the nose and mouth. Therefore, avoiding physical contact, contact through the mouth and nose, and the wearing of masks are all important defenses against transmission. The U.S. CDC also has also published research entitled: the CDC Science Agenda for COVID-19 Priority Area VI. Social, Behavioral, And Communication Science, 2020. This piece of research emphasizes the importance of effective communication within communities, empowering individuals to take appropriate measures to reduce the risks, assessment of risk communication methods and information gaps, and cultural and language-responsive information and communication tools.

In comparison to the global pandemic and the severity of its impact, the control of COVID-19 in Taiwan has been relatively good. According to data provided by the Taiwan Centers for Disease Control, the total number of COVID-19 infections and deaths in 2020 was only 785 and 7 [7], respectively, starkly contrasting to numbers from neighboring South Korea and Japan. Possible explanations include greater experience developed from the SARS outbreak, open and transparent information sharing, timely border control, advanced medical technology (From the website of the ministry of health and welfare of Taiwan, advanced medical technology includes: (1) established a green channel for rapid review with guidance from designated personnel; (2) successfully fostering the development of domestically produced test kits; (3) established a technical support platform of COVID-19 to accelerate the development of vaccines, drugs and rapid test kits; (4) collecting resources and cutting-edge technologies for the development of rapid test kits, therapeutics, and vaccine [8]), effective implementation of the central epidemic command center, adequate resource allocation, and the use of smart community epidemic prevention systems [8,9]. To effectively test for infection, Taiwan’s medical institutions first conduct rapid tests on people with a history of contact. If the rapid test result is positive, a test for nucleic acid is performed. When the nucleic acid test is confirmed to be positive, the COVID-19 patient must be isolated in the hospital. If local hospital does not have enough isolation rooms, local government can requisition hotels to become temporary epidemic prevention hotels and assign mild or asymptomatic patients to isolate there. Those with more severe symptoms will be allocated a negative pressure isolation room in the hospital.

From awareness and knowledge to implementation of epidemic prevention, these actions fall within the scope of the theory of planned behavior (TPB). Taking inspiration from [10] COVID-19 prevention measures research, we posit that perception-based factors among residents influence the prevention measures adopted. During a severe epidemic, strict control measures are often required such as the lockdown enforced in Wuhan between January to May of 2020 or the closure of restaurants, movie theaters, and other places of gathering such as in San Francisco. Under strict measures, residents are required to stay in their respective houses or community for most of the time. In Taiwan, most people live in a multiplex or single apartment building, while a small proportion live in single-family detached houses. Therefore, the focus of epidemic prevention relies on the combined action of epidemic prevention measures among residents, particularly given the commonplace congregate housing community environment.

In summary, if the overall effectiveness of the epidemic prevention relies on the epidemic prevention behaviors of residents, and the residents’ epidemic prevention behaviors are indeed affected by information and awareness relating to the epidemic, then the effective promotion of epidemic prevention information and the implementation of epidemic prevention measures are of great importance. Moreover, residents that reside in single or multiplex apartment buildings, regardless of the epidemic prevention regulations of the city, will be influenced by the immediate measures adopted from within the community.

From present studies, we know the perception of epidemic prevention is crucial to epidemic prevention [11] and what factors affect perception formation [10]. Still, we do not understand how the community’s effect on the perception of epidemic prevention. Especially under strict measures period, the community’s measures and characteristics would affect residents greater than ever before.

Therefore, it is crucial to understand whether epidemic prevention measures within a community indeed affect the awareness of epidemic prevention among residents. This study seeks to clarify the relationship between the residential community and the spread of the epidemic based on existing literature and research, and summarizes multiple factors that may affect the residents’ awareness of epidemic prevention. Additionally, using a questionnaire and quantitative methods, we clarify the impact and effect of the wider community on awareness of epidemic prevention among residents in Taiwan. The result shows that the community’s epidemic prevention measures could make residents pay more attention to epidemic prevention.

## 2. Theory and Literature

### 2.1. Housing and Health

Due to limitations in funds and technology, residential buildings built in the past were often poorly constructed. Frequently they lack sufficient space between building compartments, independent bathrooms, wastewater treatment systems, and other necessary subsistence systems. In addition, older buildings often use building materials of lower quality and lack adequate building management. Many studies have validated the relationship between housing and health. Taking building materials as an example, the use of lead pipes in water supply systems has been shown to be associated with excessive lead intake in the human body [12], as it can damage the nervous system and kidneys [13]. Humid building environments are correlated with the growth of mold, which has also been shown to be highly associated with respiratory diseases [14]. Other disease impacts related to building conditions include (but are not limited to) building temperature control, overcrowding, noise pollution, and aging buildings [15].

### 2.2. Housing and Health Problems

Housing conditions can impact the health of the occupants, as well as the spread of diseases. [16] showed that higher humidity and lower temperature increase the spread of rhinoviruses, other picornaviruses and adenoviruses. Lower humidity favors the survival and spread of parainfluenzavirus and influenzavirus A. Similar conclusions were drawn by [17].

An increasing number of studies have shown that transmission of coronavirus occurs via droplets and contact transmission [5], of which droplet transmission is the major route. Both transmissions occur over short distances [18]. Since droplet infection relies on small-particle aerosols and large-particle droplet aerosols, airborne infection characteristics are connected to the ventilation systems present inside buildings. [19] suggested that medical masks do have a protective effect on the mouth and nose following a comparison of experimental and control group. In addition, contact transmission involves surfaces on which virus are carried. Generally speaking, the surface of hard material is more porous and is more conducive to the survival and spread of the virus [20]; therefore, the contact area, material, and level of disinfection and sterilization within a building will determine the infectiousness of COVID-19.

Transmission of COVID-19 may also occur via the patients’ feces. Studies have confirmed the presence of the virus in patient feces [21]. Therefore, transmission via wastewater and fecal matter in houses or buildings is also a possibility [22]. The possibility of long-distance droplets or airborne transmission has not been fully proven. However, similar infectious diseases such as influenza, SARS and smallpox have shown to be airborne [23]. Hence, the internal ventilation system of buildings should be closely monitored. Applicable engineering control methods can include ventilation and air distribution type, air exchange rate, environmental factors (humidity and temperature) and engineering disinfection (filtration or ultraviolet irradiation), among other methods [24].

### 2.3. Household Occupants and Epidemic Prevention Awareness

The earliest research on awareness and behavior started with Fishbein’s research on psychological state and behavior [25], and forms the basis of the nascent theory of reasoned action [26]. The theory posits that human behavior is affected by behavioral intention, and an individual’s attitude influences behavioral intention and eventually leads to behavior. Because the theory of reasoned action has limitations in predicting behavior, the subsequent addition of perceived behavioral control led to the development of the theory of planned behavior (TPB). Perceived behavioral control represents the degree of an individual’s own perception on executive ability and control behavior results [27].

TPB argues that the behavior of a person is affected by behavioral intentions and perceived behavioral control. Behavioral intentions can be further subdivided into attitudes, subjective norm, and perceived behavioral control. These factors will affect the formation of behavioral intentions. Summarizing the above factors, there have been extensive empirical studies in psychology and in behavior prediction studies. The meta-analytic reviews of the TPB by [28] showed that most empirical studies demonstrated that the correlation between subjective norm and perceived behavior control is higher than 0.6, and the correlation between behavioral prediction and behavioral intention or perceived behavioral control is also between 0.4 and 0.6 [27,29,30]. Studies on drug use behavior also showed that attitude, subjective norm, and perceived behavioral control can indeed explain about 53% of behavioral prediction.

Epidemic prevention behavior is a part of entity behavior and can also be predicted using the TPB model. [10] organized and demonstrated various perceptions of epidemic prevention behaviors and showed that subjective norms, risk perceptions, epidemic prevention knowledge and attitudes, epidemic prevention promotion from the government and other factors had a positive and significant impact on epidemic prevention intention of an individual. [31]’s research on epidemic prevention behavior of young adults also showed that attitudes positively affect behavior, and subjective norms and perceived behavior control produced a significant result in the non-young-adult model.

Another research stream related to TPB is protection motivation theory (PMT). PMT studies also focus on the perception of benefit, obstacle, and health [32]. PMT and TPB both argue that the perception of epidemic prevention is crucial against the spread of Cov-19 [10,11]. They also focus on how the perception is affected by other factors or scenarios [10,33].

However, recent studies on epidemic prevention behaviors still lack observations of more detailed influencing factors such as attitudes and subjective norms on residential communities. Specifically, epidemic prevention attitudes and related awareness can indeed affect a person’s epidemic prevention behaviors, and there are also many additional relevant factors. However, the society or environment will affect the perceptions of members or residents [34]. Similarly, residential communities will also influence the formation of residents’ perceptions surrounding specific issues [35]. Therefore, when discussing awareness and attitudes towards epidemic prevention, residential communities may play a very important role. Consequently, we study the various factors that have a significant influence on the community residents’ awareness and attitudes towards epidemic prevention, and the degree of impact of various factors.

## 3. Methodology

### 3.1. Data

To test the relationship between the various conditions in the community and the residents’ awareness and attitude towards epidemic prevention, we distributed a questionnaire to collect the opinions of residents in different communities across the country on epidemic prevention awareness. The sample is the questionnaire data filled out from the Internet, there is no specific invitee for the answer to the online questionnaire. We used convenience sampling, attempting to recruit three types of respondents:

- the property manager of the housing community
- the member of the management committee
- the community household

In total, 200 valid questionnaires were collected from 18 counties and cities, which comes from most cities of Taiwan. Each questionnaire sought to capture residents’ awareness and attitudes towards epidemic prevention. The issuance and collection of the questionnaire occurred between March and September 2020. During this period, Taiwan moved from a period of strict epidemic prevention measures to conditionally resuming social activities. Most people in the country were understandably strongly impacted by the epidemic and this in turn affected attitudes towards the questionnaire.

The entire questionnaire was composed of the control conditions of individuals and communities, various epidemic prevention measures in the community, and the respondents’ attitudes towards the importance of epidemic prevention measures. The individual control conditions included age and gender. The community control conditions captured the size of the community, whether a management committee existed and whether the building was built before 1995. The size of the community was mainly classified according to the number of households. Small communities consisted of less than 149 households, medium-sized communities contained between 150 and 300 households, and 300 households or more were classified as large communities. In addition, Taiwan established new apartment building management regulations in 1995, which stipulated that all new communities must have a community management committee or a management organization. Therefore, whether the community was established before 1995 was also included. However, not all early communities have a management committee. The question also assessed the age of the community, the details are list in Appendix A.

The epidemic prevention measures implemented by the community are based on the various practices listed in the “Guidelines to COVID-19 responses: community management and maintenance” issued by the Central Epidemic Command Center of Taiwan [36], and the guidelines for community epidemic prevention measures issued by the construction and planning agency of Ministry of the Interior. Using qualitative unstructured interviews and semi-structured expert meetings to determine the autonomous epidemic prevention measures in Taiwan’s congregating housing communities, a total of 68 epidemic prevention measures were identified. For the following validation needs, we further summarized these 68 epidemic prevention measures into 12 types of epidemic prevention measures. The details are summarized in Appendix B. The statistical results of the questionnaire show that some epidemic prevention measures were adopted by most communities. For example, arrangement of dedicated epidemic prevention routes within the community. However, some epidemic prevention measures were found to be implemented by only a few communities. For example, prohibition of entry to those who refuse to wear a mask. These reflected larger differences between communities.

Our questionnaire asked residents in the community to provide responses using a Likert five-point scale, with 5 points being very important and 1 point being not at all important.

### 3.2. Variable Definitions and Sample Description

The present study hopes to improve understand of the factors influencing residents’ awareness of the importance of epidemic prevention-related practices in the community, hence the dependent variable used was the score obtained for the awareness of the importance of epidemic prevention measures, as shown in Table 1. The importance of epidemic prevention awareness can be classified into 12 types, and an additional mean importance of awareness summed up to a total of 13 types. Various types of epidemic prevention attitudes partly reflect the daily prevention habits of community residents. Every epidemic prevention method was found to be related to the community epidemic prevention effectiveness. Therefore, in the following empirical analysis the importance of awareness of these 13 categories was used as the dependent variable.

The main theoretical variables are based on community-related conditions, which are composed of three factors: the scale of the community, the establishment of a management committee, and the execution ratio of the community’s implementation of epidemic prevention measures. Community size forms the main theoretical variable, which is a continuation of previous research [37]. Since communities of different sizes will differ in the number of people entering, exiting, and gathering, there is a higher possibility of infectious disease spreads in larger communities. Moreover, large-scale communities often have more complete and rigorous management. They are more able to adopt more diverse or comprehensive epidemic prevention measures, which will also become a factor impacting the community’s vulnerability to infectious diseases and overall health [38], Therefore, this study classifies the size of the community into large, medium, and small as an important theoretical variable for examining the next step.

Another theoretical variable encodes whether the community has established a management committee. The variable takes the value of 1 denoted if a committee is established within a community, and 0 otherwise. In the early days, there existed relatively scant regulations requiring that communities establish management committees in Taiwan, hence there were many early communities lacking management teams. It was not until 1995 that “Management of apartment buildings” was enacted which mandates the establishment of management committees. This change further assured the quality of life of community residents. The management team of the community is a critical executive division which handles large and small issues in the community. The committee oversees the maintenance of important facilities in the community, the sanitation of public environment, the management of general affairs, and several services offered to residents. The epidemic prevention measures in the residential community are an important responsibility of the management committee. Therefore, the effectiveness of epidemic prevention measures between communities is likely to be impacted by the presence or absence of a management team. Based on the above reasons, the establishment of a community management committee was another important theoretical variable in our study.

The last theoretical variable is the execution ratio of the community’s implementation of epidemic prevention measures. Awareness of epidemic prevention factors can stem from self-assessed risk attitude, societal commentary, personal knowledge of epidemic prevention and social norms [10]. Social norms include the participation of practices from colleagues, peers, and family members. Regarding living conditions, epidemic prevention measures adopted by residents and preventative practices executed in the community determine the environmental and institutional atmosphere. Therefore, 68 epidemic prevention items implemented in the community were summarized in the questionnaire. We calculated the proportion of implemented measures in each community, which allowed the development of further important theoretical variables.

Other control variables included other items of the questionnaire, such as gender and age of the interviewee, whether the buildings were built before 1995, disaster prevention attitudes, attitudes toward community organizations, and whether interviewees were in the Greater Taipei area. A binary variable for gender was used which took the value of 1 for males, and 0 otherwise. Age was divided into three ranges, the first range was 18 to 44 years old, the second range was 44 to 65 years old, and the third range was 65 years old and above. “Management of apartment buildings” were enacted in Taiwan in 1995. Prior to 1995, there were very few management committees in the community. According to data from the Taipei City construction management office, as of the end of 2020, only 2941 had established management teams and reported to the government with a building use license in a 7 or more-storied building built before 1995. The filing rate was 36.12%. From 1995 to 2004 buildings with use license had a filing rate of 78.3%, which grew to 90.25% after 2004. However, as described an important factor was whether the community management committee was established or not. Therefore, this variable also captures the housing age of the residential community. Communities built before 1995 are more than 25 years old. Communities formed after 1995 represent relatively young buildings.

There were two factors that demonstrated the awareness of the importance of general disasters and the importance of the general community operation determined by backward selection from 68 kinds of questionnaire question. (1) The community’s disaster prevention attitude that “reserves a certain amount of edible water and fast food for emergency use by service personnel”, and (2) the attitude towards community management operations that “pays attention to the protection of funds such as community management fees and the cash flow of public funds”. Additionally, implementation of the epidemic prevention in the community also encompasses disaster response, another responsibility of the community management team. Therefore, we speculate that the two factors above may also affect residents’ perception of the importance of epidemic prevention measures. Hence, they too, are added as control variables.

The questionnaire asked if the interviewee lives in the Greater Taipei area which included communities located in Taipei City or New Taipei City. The variable took the value of 1 if the interviewee was in one of these two cities, and 0 otherwise. As Taipei City is the capital of Taiwan, it has the most industrial investment and the highest population density in the country. With the growth of urban development trend, Taipei City’s influence has gradually expanded to the neighboring New Taipei City area. New Taipei City became the living place for many laborers and practice of commuting to work in Taipei City increased. Greater Taipei area has become the most populous metropolis in Taiwan. Residents living in large metropolitan areas often have different attitudes towards epidemic prevention from those living in small cities or rural areas with a smaller population [10]. Therefore, this study also considered whether respondents lived in the Greater Taipei area as one of the control variables.

### 3.3. Econometric Model

The dependent variables used in this study were the mean value of a five-point scale. Therefore, the minimum value was one and the highest value could not exceed five and exhibited typical data truncation characteristics. Given the dependent variable ranged from one to five and showed the characteristics of a continuous variable, traditional OLS was used for the empirical model. However, as the model does not specifically adjust for truncated variables, this raised the risk of obtaining larger errors [39]. Therefore, a truncated regression model was used in this study (We also use a logistic regression model to test. However, the actual test results show that there is not much difference in the significance of variables, but explanatory power is lower than the truncated regression model). Estimation was calculated using the following formula:

$$y_{i}=\beta_{0}+\Sigma \beta_{i}X_{i}+\Sigma \gamma_{i}Z_{i}+\varepsilon_{i}$$

where $y_{i}$ is the dependent variable of the model, $X_{i}$ is the independent variable, $\beta_{i}$ is the coefficient of the independent variable, $Z_{i}$ is the control variable, $\gamma_{i}$ is the coefficient of the control variable, $\beta_{0}$ is the constant term of the model, and $\varepsilon_{i}\;$ is the random error.

## 4. Empirical Results

### 4.1. Sample Analysis and Descriptive Statistics

As Taiwan’s communities scale are relatively small, households usually have a relatively high degree of understanding of the community. Furthermore, this questionnaire is not distributed to a specific minority of invitees, and there are many questions in this questionnaire. If they do not know enough about the community, it is difficult to answer all the content thoroughly. Therefore, we could observe from the result of the questionnaire. The result also shows all the questions filled, reflecting a higher degree of understanding of the community. Basically, we could compare the sample’s distribution with the distribution of houses unit in Taiwan. The house number data are from the population and housing census in 2010. From Table 1, we can observe that there is not obvious distribution difference between the sample and cities in Taiwan.

The indicator to exam the reliability of this questionnaire is Cronbach’s alpha. We perform it in Table 2, which shows all the perception is greater than 0.8, represents the result of the questionnaire is reliable. The mean of the overall questionnaire showed that most of the community respondents were very familiar with issues related to epidemic prevention. There was a high awareness of the importance of epidemic prevention measures, the statistical results of which are summarized in Table 2. The epidemic prevention importance awareness of all categories had a mean of 4.239 points, indicating that most of the items were highly valued by residents. Other items also showed higher awareness of the importance in measures. Among them, the highest mean score of all categories was epidemic prevention measures, with an average value of 4.416 points. The lowest was the use of smart epidemic prevention measures systems, with a mean of 3.915 points, which is close to a score of 4 points on the scale.

The narrative statistics of various variables can be observed from Table 3. It was shown from the independent variables that the size of the community was mainly small and medium-sized, and the mean number of management committees established did not exceed 0.5. This also demonstrated that there were many communities lacking management teams, and the implementation rate identified was only 0.318. On average, the implementation of epidemic prevention measures was only in a little more than 30%, and nearly 70% were not implemented.

After confirming the characteristics of the sample and the narrative statistics, we can now start to observe the focus of our study, i.e., the awareness of the importance of epidemic prevention. To understand whether the awareness of the importance of epidemic prevention among community residents differs due to the scale of their respective community, we first performed a basic paired *t*-test to compare whether there were significant differences between three different community sizes, the results are shown in Table 4. When we use paired *t*-test, some studies may consider using corrections for multiple comparisons (e.g., Bonferroni correction). The paired sample *t*-test was conducted in Table 4, which may have a type I error enlargement problem. For such a situation, we also reviewed some literature recommendations for this situation. For example, [40] also mentions such a situation. Still, he also argues that the traditional Bonferroni correction method will also lead to over acceptance of a false null hypothesis, and the probability of type II error is increased. After considering the above factors, we still present the contents of Table 4.

In the table, it was clear that most of the importance awareness did not differ due to the size of the community. Medium-sized communities attached less importance to perception teach and perception surrounding than smaller communities. Larger communities attached less importance to perception prohibit than smaller communities. Large-scale communities attached less importance to perception equipment and perception admin than medium-sized communities. In addition, we also conducted a paired *t*-test between committee and execution ratio. Most results were found not to be significant. Therefore, to evaluate the impact of the independent variables on the awareness of the importance of epidemic prevention, we included the aforementioned independent and control variables and performed an empirical test.

### 4.2. The Empirical Model Result

To ensure that the empirical model did not have a high degree of collinearity, we produced a matrix of correlation coefficients first, the results are shown in Table 5. If we use 0.8 as a high correlation standard, we show that there were no variables that exhibited a high degree of correlation. Therefore, we tested the impact of various theoretical variables in the empirical model.

The empirical results of various awareness attitudes on the importance of epidemic prevention are shown in Table 6. This included the aforementioned 12 different epidemic prevention items and a comprehensive mean awareness of the importance of epidemic prevention. The estimated results of a total of 13 models are listed in Table 6. On the left of the column, the variables are listed. The first row shows the name of models, and the dependent variables were the various epidemic prevention categories. The value above the brackets in the column show the estimated coefficient of the model, and the value in the brackets is the t-statistic converted into a *p*-value.

From the table, it can be seen that in most of the epidemic prevention attitude models, people who originally paid more attention to general affairs and the disaster prevention preparations also attached more importance to epidemic prevention measures. In particular, the community operation variable showed a significant positive effect in various models. Except for Model 9 that had a significance level of 0.1, the remaining models showed a significance at the level of 0.01. This demonstrates that residents who actively participate in community general affairs appear to be more attentive during the epidemic prevention period. Material reserve also showed a significant positive impact in most models, except for the three models which were Model 8, Model 12, and Model 13. This also reflected the fact that people who paid more attention when preparing for disasters during normal times, were also more aware of epidemic prevention measures.

The three main independent variables did not show significance in most empirical models. Scale had no obvious difference and impact in most models. However, it was found to be significant in the Model 12. This was similar to the *t*-test results, except that the coefficients identified were of opposite sign. However, the empirical model at this stage has already controlled for many variables. Hence, we believe the results are reliable and reflect the fact that residents of larger communities attach more importance to entry restrictions. Committee also showed insignificant results in most of the models but showed a negative effect in the *p* follow model indicating that with a community management committee, residents counterintuitively paid less attention to related tracking and monitoring measures upon entry. Lastly, the E ratio also showed insignificant results in most models but showed a positive effect in the Model 3. This indicates that the rate of the implementation of epidemic prevention measures caused residents to be more aware of information about epidemic prevention and related measures.

Among other control variables, residents in communities built before 1995 also had a greater awareness of the importance of epidemic prevention measures, and a lower awareness of the importance of entry restrictions in the community. Older communities paid less attention to disease tracking than newer residential communities. Great Taipei also exhibited a positive impact in the Model 3and Model 13, which confirms that residents of the metropolitan areas have a higher awareness and vigilance of towards the epidemic. Age showed no significant impact in most models, but was positively correlated with Model 12, demonstrating that older people attach greater importance to people who do not follow appropriate epidemic prevention measures upon entry to the community.

The above empirical analysis results can only be used in a few cases to verify the hypothesis of the role of the independent variables, and it fails to show the expected result as the content of the literature and pre-existing theories above—however, these aspects of the current research design that could be assessed in more details. The regression summarized in Table 6 showed that the important theoretical variables, scale, and committee, were pooling data applied to the empirical model. Only dummy variables were used to control the changes of these two independents variables. This may not have been sufficient to identify the difference between the scale of community and the effect of a management committee. Therefore, to assess the difference in more detail, we performed a further regression analysis on scale and committee variables. The type of model was based on the mean of importance awareness. Because the most direct impact on the effectiveness of epidemic prevention was implementing epidemic prevention measures and the entry restriction, Model 1, Model 5, and Model 12 were used in advanced empirical models. The results are summarized in Table 7 and Table 8.

It is shown in Table 7 that the significance of the main independent variables changed. Compared with Table 6, Committee was originally only significant in Model 13, but in Table 7 it was significant in the Model 5 for medium scale community and Model 12 for the small and large community models. This allowed us to observe model differences in greater detail. From the statistical results, committee was found to have a negative impact on epidemic prevention-related measures in medium-sized communities, indicating that the residents of a medium-sized community paid less attention to epidemic prevention-related measures in the presence of a management committee. However, in the entry restriction to the community model, committee had two completely different effects. In a small community, the establishment of a management committee reduced residents’ awareness of the importance of entering the community without appropriate preventative measures. Conversely, in a large community, the presence of a management committee increased the awareness of the importance of entering the community without appropriate preventative measures.

E ratio was found to be significant in three models: Model 1, Model 5, and Model 12. Model 1’s dependent variable was the mean of awareness of the importance of all epidemic prevention-related categories, the empirical results in Table 7 showed the mean effect of epidemic prevention of the three different community scales. The E ratio showed a negative effect on Model 1 in medium and large-scale communities. In medium scale community, E ratio increased therefore increasing residents’ awareness of the overall importance of epidemic prevention, but awareness decreased in large-scale communities. This phenomenon was also observed on Model 5 in the medium and large-scale community. With the increase in the implementation rate of community epidemic prevention measures, residents’ awareness of the importance of epidemic prevention measures increased in medium-sized community but decreased in larger communities. Residents were found to have lower awareness of the importance of epidemic prevention measures. In the Model 12, only large-scale community showed significance, indicating that in such communities the increase in the implementation rate of community epidemic prevention measures increased the awareness of the importance of improper prevention measures upon entering the community.

Among other control variables, residential communities built before 1995 had similar effects to E ratio in Model 1 and Model 5, which were found to have a positive effect in medium-sized communities, but a negative effect in large-sized communities. The Model 12 showed negative impact in small and medium-sized communities but a positive impact on large communities. The Great Taipei variable only showed significance in large communities, and the results were found to be consistent with the positive impact reported in Table 6. The impact of the Greater Taipei was slightly higher than the coefficient regardless of the size of the community. The location had a positive impact on the awareness of the importance of epidemic measures in large communities, and a negative impact on the prohibition of entry into the community without appropriate epidemic prevention. Community operation and material reserve also showed a positive effect in most models, but in Model 12, community operation had a negative effect on large communities. This was inconsistent with the results shown before. Age showed clear differences with the classification of community size. In Model 12, the increase in the age of residents in small communities reduced the awareness of the importance of entering the community without appropriate epidemic prevention measures. However, in large communities, increase in age of residents increased and enhanced the awareness of its importance. This was consistent with the aforementioned empirical results.

Table 8 demonstrated the truncated regression model results following the classification of community management committees. The scale variable was only significant in Model 12, which showed that as the size of community expanded and in the absence of a community management committee, residents showed reduced awareness of prohibition measures in relation to people entering the community without having taken appropriate precautions. In the presence of a committee, the results were not significant. When a management committee was present, E ratio, the rate of implementation of epidemic prevention measures, increased residents’ awareness of the importance of epidemic prevention measures. The Model 12 showed that regardless of the presence of a management committee, implementation rate of epidemic prevention measures promoted the awareness of the importance of appropriate epidemic prevention measures upon entering the community.

Communities formed before 1995 showed a negative impact in the presence of a management committee in Model 12. This was consistent with the results shown in Table 6 and Table 7. In Model 12, the Great Taipei variable had a negative impact regardless of the presence of a committee, which was consistent with the results of shown previous. However, negative impact was even greater without a management committee. Operation and reserve presented a positive effect in the model, which was mostly consistent with the results shown previously. Age also showed a positive effect in Model 5 without a management committee and in Model 12 in the presence of a management committee.

## 5. Discussion

Through the empirical analysis at each step, we confirmed that people are indeed affected by environmental factors. The awareness factor was one of the principally affected aspects. This was the focus of this article. If we can effectively change awareness factors, behavioral intentions can be influenced, and this will ultimately shape behavior. Epidemic prevention behavior is formed through epidemic prevention intention and attitude. Hence, the attitude and related awareness of epidemic prevention is critical. From current research, we find that the awareness factors of epidemic prevention directly affect the epidemic prevention intention of an individual [10]. A further question is what are the factors affecting attitudes to or awareness of epidemic prevention?

To answer the above issue, this article used a questionnaire as the basis of empirical research in Taiwan. A detailed interview was conducted on community conditions and measures related to epidemic prevention. Epidemic prevention implementation in each community and residents’ awareness of the importance of epidemic prevention was analyzed. Table 2 showed that residents in most communities scored 4 points or more for the awareness of the importance of epidemic prevention. This may be due to past epidemic prevention promotion and the 2003 SARS epidemic experience in Taiwan. The general population attached great importance to epidemic prevention.

From the results of the questionnaire, we hoped to understand whether the relevant measures and conditions of a community affected the residents’ awareness of epidemic prevention. We first performed a paired *t*-test to observe whether epidemic prevention attitudes differed due to the size of the community. Based on the results in Table 4, the overall difference was found to be modest, and only a small part of the awareness of the importance of epidemic prevention differed due to the size of the community. Therefore, before discussing the impact of variables in more detail, the size of the community was judged to not be an important factor. Additionally, the committee and execution ratio variables did not show too many significant results with the paired *t*-test.

The results of the first stage of our analysis provided negative answers to the questions that were posed. Yet to better understand the impact of the difference in community conditions more carefully, we performed a second- and third-stage regression analysis. The second analysis used 13 markers of awareness of importance of epidemic prevention as dependent variables. The empirical results in Table 6 showed that independent variables did not have a significant impact in most models, and only a few showed significances. For example, the E ratio increased the perception of importance in Model 3, and the expansion of community scale also increased perception of importance in Model 12. In Model 13, the committee variable decreased the perception of importance. Overall, the results support few theoretical expectations.

The third stage regression analysis uncover the E ratio’s positive effect on the perception of importance deeply. From Table 7 and Table 8, more influence of E ratio was found in more models. Model 1 and Model 5 showed that the impact of E ratio increased in medium-sized communities but showed a negative impact in large communities. Model 12 only showed an improvement of the impact on large-scale communities. Therefore, from the perspective of the community scale, E ratio appears to influence residents’ awareness of the importance of epidemic prevention. It may have few negative effects in certain aspects in large communities. However, increasing the E ratio of the community can still effectively prevent the spread of the epidemic. The committee variable in Table 7 did not show consistent significance. Table 8 showed that in some important respects, the presence of a committee, meant that the E ratio increased and demonstrated a positive effect. The scale variable only showed a negative impact under the no committee condition.

From the overall empirical results, the larger the community, the more susceptible the residents to measures in the community and the more that resident was affected awareness. Large community naturally have more people who may gather. This is conducive to the spread of the virus. Hence such a community might adopt more epidemic prevention measures, and these are necessary from the perspective of epidemic prevention effectiveness or raising residents’ awareness of epidemic prevention. Although the presence of a committee may not be significant in all cases, under certain important awareness conditions, the E ratio increased, and therefore helped to improve residents’ awareness of the importance of epidemic prevention in the presence of a committee. From the perspective of operation, the presence of a committee makes operations more effective in improving residents’ awareness of the importance of epidemic prevention. This may stem from the mutual relationship between committee and operation. Funding of community organizations may be related to the projects implemented by management committees. It is more difficult to observe the use of funds in communities lacking management committees, hence, naturally people are likely to be less concerned. Residents who pay attention to disaster prevention preparations show that they are also attached to epidemic prevention-related matters, which is consistent with their interest in caring about day-to-day community operations.

## 6. Conclusions

The main contribution of this study is to show the characteristics of a community could affect residents’ awareness of epidemic prevention, which answers the question in the introduction and supplementing the role of community in epidemic prevention’s perception studies. Stated differently, the management committee could affect residents’ perception indirectly; owning from the community’s implementation of epidemic prevention measures could affect residents’ perception directly.

Another significant contribution of this study is to provide suggestions for future community epidemic prevention. The first thing that can be implemented is that for residential communities that have not yet established a community management organization or the management committee, the local government could guild the community to establish a management organization. Once the management organization is established, the necessary epidemic prevention measures can be implemented in accordance with the needs of the community, and community residents should also be encouraged to pay attention to the preparation for general disasters and the implementation of various community affairs. The promotion of these matters will directly or indirectly help make community residents have a more serious attitude toward epidemic prevention.

The main research limitation of this article comes from the questionnaire design and questionnaire respondents. Because the online questionnaires distributed in this article are filled by property managers, management committee members, and residents, the residents’ responses may cause some errors in the actual implementation of the project in the housing community. Moreover, we have not used the stratified random sampling to distribute online questionnaires, which also makes the sample in this study not sufficiently random. The second limitation of the questionnaire is that we do not design questions to fill in household income, education, and price or rent information in housing communities. These variables may also affect attitudes related to epidemic prevention and ultimately may affect the awareness of the importance of epidemic prevention. The weakness of this study is also the start point for the following studies.

In addition to the above limitations, the questionnaire results of this study are still consistent and reliable, and the results of the study are using appropriate econometric methods. For the current epidemic prevention research literature, conclusions of this study can still provide for scholars or local governments.

## Figures and Tables

**Table 1:** The distribution difference between the sample and cities’ house numbers in Taiwan.

City	House Unit	Ratio (A)	Sample	Ratio (B)	A−B
NewTaipei	1,500,783	18.9%	22	11.0%	7.9%
Taipei	921,564	11.6%	34	17.0%	−5.4%
Keelung	162,822	2.0%	6	3.0%	−1.0%
Hsinchu	157,082	2.0%	4	2.0%	0.0%
Yilan	161,234	2.0%	6	3.0%	−1.0%
Taoyuan	738,111	9.3%	20	10.0%	−0.7%
Taichung	947,713	11.9%	24	12.0%	−0.1%
Miaoli	165,349	2.1%	6	3.0%	−0.9%
Changhua	388,202	4.9%	6	3.0%	1.9%
Nantou	160,686	2.0%	0	0.0%	2.0%
Yunlin	214,558	2.7%	6	3.0%	−0.3%
Tainan	645,134	8.1%	16	8.0%	0.1%
Kaohsiung	1,003,089	12.6%	24	12.0%	0.6%
Chiayi	275,829	3.5%	6	3.0%	0.5%
Pingtung	270,884	3.4%	6	3.0%	0.4%
Penghu	29,365	0.4%	1	0.5%	−0.1%
Taitung	74,716	0.9%	6	3.0%	−2.1%
Hualien	121,344	1.5%	6	3.0%	−1.5%
Kinmen	15,647	0.2%	1	0.5%	−0.3%

**Table 2:** Statistical results on the awareness of the importance of epidemic prevention categories.

Perception	Mean	SD	Cronbach’s Alpha
Average	4.239	0.289	-
Living assistance measures during the epidemic prevention period	4.333	0.438	0.908
Epidemic prevention promotion	3.983	0.416	0.897
Epidemic prevention configuration	4.175	0.471	0.902
Ordinary epidemic prevention measures	4.416	0.292	0.895
Epidemic prevention teaching and training	4.245	0.480	0.904
Community facilities and environmental disposal management during the epidemic prevention period	4.364	0.466	0.908
Epidemic prevention equipment	4.410	0.373	0.904
Epidemic prevention and care	4.158	0.423	0.904
Community epidemic prevention administrative management	4.327	0.454	0.902
Smart epidemic prevention systems	3.915	0.552	0.899
Entry restriction	4.370	0.490	0.903
Entry tracking	4.173	0.515	0.906

Notes. SD = standard deviation.

**Table 3:** Descriptive statistics.

Variables	Mean	SD	Max	Min
Theory variables				
Scale	1.935	0.815	3	1
Committee	0.450	0.499	1	0
Execution ratio	0.318	0.284	1	0
Control variables				
Male	0.495	0.501	1	0
Age	1.710	0.761	3	1
Before 1995	0.615	0.488	1	0
Material reserve	4.440	0.699	5	1
Community operation	4.020	0.716	5	2
Great Taipei	0.280	0.450	1	0

**Table 4:** Paired *t*-test of the awareness of the importance of epidemic prevention and community scale.

Community Scale	1 vs 2		1 vs 3		2 vs 3	
Awareness	Ha < 0	Ha > 0	Ha < 0	Ha > 0	Ha < 0	Ha > 0
Average	0.347	0.653	0.076	0.924	0.120	0.880
Living assistance	0.805	0.195	0.585	0.415	0.249	0.752
Propaganda	0.881	0.119	0.522	0.478	0.130	0.871
Distribution	0.385	0.610	0.219	0.782	0.303	0.697
Ordinary measures	0.176	0.824	0.087	0.913	0.315	0.685
Teaching and training	0.027 **	0.973	0.148	0.852	0.802	0.198
Community facilities	0.008 ***	0.992	0.205	0.795	0.924	0.076
Equipment	0.423	0.577	0.039	0.962	0.044 **	0.956
Care	0.869	0.131	0.155	0.845	0.008	0.992
Administration	0.863	0.137	0.056	0.945	0.006 ***	0.994
Smart systems	0.078	0.922	0.117	0.883	0.628	0.372
Entry restriction	0.062	0.938	0.002 ***	0.998	0.081	0.919
Entry tracking	0.438	0.562	0.323	0.677	0.353	0.647
observations	1 = 73	2 = 67	1 = 73	3 = 60	2 = 67	3 = 60

Note: The value in the column denotes the *p*-value after the conversion of the *t*-test value, in which the significance level is 95%, ** represents the *p*-value less than 5%, *** represents the *p*-value less than 1%. The sequence of first column is equal to the first column of Table 2. The number in first row represents different scale of community, 1 is small community, 2 is medium community, and 3 is large community. Ha refers to alternative hypothesis.

**Table 5:** Correlation coefficients of independent variables.

Variable	Male	Age	Scale	Committee	1995	Greattaipei	Operation	Reserve	Eratio
male	1.000								
age	−0.083	1.000							
scale	−0.105	0.213	1.000						
committee	0.130	−0.184	−0.039	1.000					
1995	−0.203	0.090	−0.089	−0.689	1.000				
Greattaipei	0.051	−0.173	−0.197	0.152	0.013	1.000			
Operation	−0.065	0.147	0.095	0.078	−0.046	−0.074	1.000		
Reserve	−0.182	0.122	0.045	0.017	0.065	−0.080	0.103	1.000	
Eratio	0.151	−0.159	0.118	0.638	−0.647	0.086	−0.071	−0.064	1.000

**Table 6:** Regression analysis of the awareness of the importance of epidemic prevention.

Variable	Model 1	Model 2	Model 3	Model 4	Model 5	Model 6	Model 7	Model 8	Model 9	Model 10	Model 11	Model 12	Model 13
Male	0.045	0.142 **	0.119 **	0.049	0.028	−0.029	0.134 **	0.030	0.001	−0.006	0.050	−0.024	0.032
	(0.225)	(0.022)	(0.028)	(0.456)	(0.489)	(0.662)	(0.041)	(0.556)	(0.984)	(0.922)	(0.493)	(0.725)	(0.660)
Age	0.018	0.053	0.009	0.028	0.030	−0.064	−0.027	0.013	−0.032	0.066	0.040	0.103 **	0.005
	(0.457)	(0.201)	(0.798)	(0.536)	(0.268)	(0.149)	(0.541)	(0.718)	(0.412)	(0.112)	(0.413)	(0.022)	(0.913)
Scale	0.017	−0.018	−0.003	0.023	0.013	0.044	0.028	0.036	0.041	0.002	0.013	0.075 *	0.004
	(0.466)	(0.640)	(0.922)	(0.587)	(0.613)	(0.294)	(0.501)	(0.264)	(0.261)	(0.949)	(0.782)	(0.075)	(0.930)
Committee	−0.015	0.047	0.018	0.059	−0.038	0.020	−0.086	−0.028	0.128	−0.119	−0.012	−0.031	−0.181 *
	(0.784)	(0.612)	(0.820)	(0.550)	(0.530)	(0.840)	(0.380)	(0.711)	(0.141)	(0.193)	(0.914)	(0.759)	(0.091)
1995	0.022	0.134	0.143 *	0.014	0.018	0.055	−0.075	−0.098	0.141	−0.039	0.058	−0.065	−0.265 **
	(0.695)	(0.151)	(0.078)	(0.892)	(0.762)	(0.578)	(0.449)	(0.207)	(0.109)	(0.673)	(0.595)	(0.520)	(0.015)
GreatTaipei	0.023	0.003	0.193 ***	0.060	−0.026	−0.002	0.007	−0.001	0.003	−0.071	−0.073	−0.019	0.148 *
	(0.574)	(0.970)	(0.001)	(0.422)	(0.562)	(0.977)	(0.928)	(0.990)	(0.965)	(0.303)	(0.373)	(0.796)	(0.067)
Operation	0.165 ***	0.120 ***	0.197 ***	0.165 ***	0.122 ***	0.157 ***	0.149 ***	0.153 ***	0.073 *	0.154 ***	0.244 ***	0.182 ***	0.184 ***
	(0.000)	(0.007)	(0.000)	(0.000)	(0.000)	(0.001)	(0.001)	(0.000)	(0.079)	(0.000)	(0.000)	(0.000)	(0.000)
Reserve	0.113 ***	0.074 *	0.130 ***	0.063	0.061 **	0.161 ***	0.103 **	0.020	0.162 ***	0.148 ***	0.211 ***	0.011	0.068
	(0.000)	(0.084)	(0.000)	(0.169)	(0.028)	(0.000)	(0.023)	(0.571)	(0.000)	(0.001)	(0.000)	(0.818)	(0.176)
E ratio	0.103	0.064	0.298 **	0.002	0.106	0.012	0.105	0.060	−0.077	0.122	0.221	0.093	−0.018
	(0.248)	(0.669)	(0.023)	(0.988)	(0.277)	(0.939)	(0.509)	(0.634)	(0.586)	(0.417)	(0.213)	(0.568)	(0.920)
Cons	2.923 ***	3.255 ***	2.273 ***	3.021 ***	3.520 ***	2.893 ***	3.264 ***	3.597 ***	3.036 ***	2.995 ***	1.788 ***	3.24 ***	3.261 ***
	(0.000)	(0.000)	(0.000)	(0.000)	(0.000)	(0.000)	(0.000)	(0.000)	(0.000)	(0.000)	(0.000)	(0.000)	(0.000)
Wald chi^2^	71.31	19.5	59.34	19.21	31.42	30.55	21.5	27.75	29.45	38.89	50.96	34.48	37.73
*p* > chi^2^	0.000 ***	0.02 **	0.000 ***	0.02 **	0.000 ***	0.000 ***	0.01 **	0.000 ***	0.000 ***	0.000 ***	0.000 ***	0.000 ***	0.000 ***
Obs	200	200	200	200	200	200	200	200	200	200	200	200	200

Note: The value above the bracket was the estimated coefficient. The value inside the bracket was the *p*-value. *, **, *** denoted significance level of 0.1, 0.01 and 0.001, respectively. The dependent variable’s sequence of Model 1–13 is equal to the sequence of first column in Table 2. Cons refers to constant.

**Table 7:** Truncated regression model of different scales of a community.

Variables\Scale	Model 1			Model 5			Model 12		
	Small	Medium	Large	Small	Medium	Large	Small	Medium	Large
Male	0.019	0.066	0.066 *	0.007	0.054	0.030	0.089	−0.126 *	−0.021
	(0.785)	(0.259)	(0.093)	(0.934)	(0.392)	(0.524)	(0.242)	(0.095)	(0.503)
Age	0.000	0.060	−0.021	0.025	0.046	−0.010	−0.130 *	0.061	0.065 ***
	(0.997)	(0.138)	(0.385)	(0.729)	(0.292)	(0.731)	(0.089)	(0.322)	(0.002)
Committee	−0.020	−0.047	0.163	−0.037	−0.220 *	0.165	−0.323 ***	−0.230	0.235 **
	(0.815)	(0.681)	(0.101)	(0.737)	(0.066)	(0.171)	(0.001)	(0.306)	(0.021)
1995	0.062	0.181 **	−0.267 **	0.040	0.194 **	−0.198 *	−0.275 ***	−0.197 *	0.257 ***
	(0.511)	(0.021)	(0.003)	(0.744)	(0.021)	(0.065)	(0.000)	(0.092)	(0.000)
GreatTaipei	0.005	−0.135	0.218 ***	−0.098	−0.079	0.190 ***	−0.007	−0.114	−0.177 **
	(0.946)	(0.138)	(0.000)	(0.281)	(0.411)	(0.001)	(0.929)	(0.231)	(0.022)
Operation	0.244 ***	0.150 ***	0.049	0.213 ***	0.066	0.029	0.086	−0.043	−0.042 *
	(0.000)	(0.001)	(0.114)	(0.000)	(0.154)	(0.426)	(0.110)	(0.467)	(0.062)
Reserve	0.150 ***	0.148 ***	0.034	0.053	0.117 **	0.026	0.085	−0.079	0.027
	(0.002)	(0.001)	(0.177)	(0.395)	(0.017)	(0.378)	(0.202)	(0.168)	(0.200)
E ratio	0.155	0.409 ***	−0.574 ***	0.112	0.701 ***	−0.516 ***	−0.009	0.274	0.816 ***
	(0.260)	(0.025)	(0.000)	(0.529)	(0.000)	(0.001)	(0.972)	(0.316)	(0.000)
Wald chi^2^	44.06	43.74	61.74	18.22	29.77	24.84	23.35	16.52	108.4
*p* > chi^2^	0.000 ***	0.000 ***	0.000 ***	0.019 ***	0.002 ***	0.001 ***	0.002 ***	0.035 **	0.000 ***
Obs.	73	67	60	73	67	60	73	67	60

Note: The value above the bracket was the estimated coefficient. The value inside the bracket was the *p*-value. *, **, *** denoted significance level of 0.1, 0.01 and 0.001, respectively.

**Table 8:** Truncated regression model of the presence of a management committee.

Variable	Model 1		Model 5		Model 12	
	No	Exist	No	Exist	No	Exist
Male	0.049	0.077	0.048	0.041	0.122	−0.106
	(0.149)	(0.264)	(0.279)	(0.579)	(0.283)	(0.113)
Age	0.036	−0.005	0.065 **	−0.030	0.040	0.138 ***
	(0.103)	(0.917)	(0.024)	(0.587)	(0.623)	(0.004)
Scale	0.005	0.035	0.002	0.041	−0.112 *	−0.036
	(0.804)	(0.458)	(0.953)	(0.416)	(0.084)	(0.440)
1995	−0.014	0.050	−0.095	0.089	0.049	−0.258 ***
	(0.818)	(0.573)	(0.248)	(0.348)	(0.765)	(0.006)
GreatTaipei	0.015	0.030	−0.073	0.046	−0.419 **	−0.230 ***
	(0.749)	(0.690)	(0.230)	(0.572)	(0.013)	(0.003)
Operation	0.099 ***	0.278 ***	0.061 **	0.236 ***	−0.036	−0.020
	(0.000)	(0.000)	(0.035)	(0.000)	(0.586)	(0.721)
Reserve	0.009	0.226 ***	−0.051	0.182 ***	−0.109	−0.083 *
	(0.698)	(0.000)	(0.103)	(0.000)	(0.184)	(0.076)
E ratio	−0.056	0.136	−0.116	0.313 *	2.402 ***	1.403 ***
	(0.621)	(0.372)	(0.429)	(0.055)	(0.000)	(0.000)
Wald chi^2^	30.12	59.32	21.13	37.48	39.09	119.88
*p* > chi^2^	0.000 ***	0.000 ***	0.006 ***	0.000 ***	0.000 ***	0.000 ***
Obs.	110	90	110	90	110	90

Note: * *p* < 0.05, **p < 0.01, *** *p* < 0.001.

## Data Availability

The data presented in this study are available on request from the corresponding author. The data are not publicly available in compliance with the investigation confidential.

## Appendix A

**Table A1:** The Community Epidemic Prevention Questionnaire.

No.	Question Content	Question Format Rating	Execution (Yes/No)
		1 Not at All Important 2 3 4 5 Very Important	
1	Community independently establishes epidemic prevention checklists.		
2	Improve epidemic prevention management rules and standard operating procedures (SOP).		
3	Members of the management committee or residents share the work of epidemic prevention, and establish an epidemic prevention response team.		
4	Put masks, thermometers, gloves, alcohol, disinfectant, dry cleaning gel, goggles or face masks as needed, and manage epidemic prevention supplies well.		
5	Pay attention to community funds such as community management fees and public fund account cash flow.		
6	Provide medical supplies for front-line property management staff or provide more encouragement and substantial rewards during the epidemic.		
7	Conduct epidemic prevention exercises at the right time to facilitate the establishment of epidemic prevention concepts and disaster response actions. (Like a fire drill).		
8	Record travel history, contact history and health record.		
9	Daily self-health check: test body temperature, put a sticker or badge to show that you are all right.		
10	Wear masks, face masks, gloves and other necessary protective supplies for daily work.		
11	Place protective supplies including disposable medical surgical masks, gloves, and isolation plates.		
12	Check and monitor the performance of temperature testing equipment such as forehead thermometer and infrared rays.		
13	Store a certain amount of edible water and fast food for emergency use by community service staff.		
14	Plan service areas and home delivery storage areas, to reduce chances for residents to contact community service staff.		
15	Disinfect and clean community service staff’s workplaces daily.		
16	Change of air in workplaces. Have windows open as much as possible, and adopt natural change of air requirements.		
17	Ask the appointed management company and related third-party vendors propose corresponding management methods during epidemic prevention period in the community.		
18	Set emergency plans for suspected infection of community service staff: emergency notification, quarantine, and treatment in accordance with relevant government regulations.		
19	Related check forms and control measures are easy to understand, learn, and do.		
20	Conduct epidemic prevention and control training for community service staff.		
21	Establish a communication list of external support units such as hospitals, district chiefs, public institutions, and volunteer groups.		
22	Establish an epidemic prevention care team to actively care and understand the disadvantaged households in the community and surrounding neighborhoods.		
23	Provide relevant assistance to economically disadvantaged households whose livelihoods are affected due to the epidemic to stabilize them.		
24	During quarantine, domestic violence cases are prone to occur, and households are encouraged to strengthen self-protection. Management committees and community workers enhance attention and care.		
25	Recruit short-term care workers in the community to help reduce life difficulties for the elderly, and initiate short-term care workers mechanism.		
26	Take the initiative to care for the hearing impaired and visually impaired in the community, and assist in obtaining information so that they can receive the latest information in time.		
27	For those who have been listed living alone and have limited mobility, assist government unit in distribution of care masks.		
28	Convert complex and difficult community control procedures into easy-to-read and easy-to-understand contents.		
29	Comply with government policies. Carry out necessary control or help with those who refuse home quarantine, in accordance with community regulations.		
30	During quarantine, communication and coordination should be enhanced to settle disputes among households.		
31	Assist households in home quarantine and independent health management, such as food delivery, ensuring normal operation of water, electricity, gas, and other home supplies.		
32	Maintain facilities such as water, electricity, gas, Internet, garbage disposal, air conditioning systems, sewage disposal, toilets, and other facilities in advance to ensure that they are operational.		
33	Public areas such as indoor children’s playrooms, multi-functional activity spaces, the elderly’s classrooms, chess rooms, etc. are temporarily closed.		
34	Hand cleaning and disinfection equipment should be placed in all unclosed public facilities for use.		
35	Service counter, entrance, and exit are equipped with cleaning fluid for people entering and exiting.		
36	The elevator control panel has stickers which is replaced daily. Elevator equipment has cleaning and maintenance records.		
37	Keep complete records of environmental disinfection and cleaning (regularly check hand cleaning supplies in public areas, and refill at any time).		
38	Garbage area is timely cleaned without stacking.		
39	Cleaning staff work strictly in accordance with specified work standards. For example, you must wear gloves and masks throughout work process, and avoid accidental touch with mouth, nose, and eyes, or splashes of detergent.		
40	Garbage generated during home quarantine shall be collected and processed by professional agencies.		
41	Enhance frequency of outdoor public space disinfection, ditch cleaning, tree pruning, water tanks cleaning, and other environmental cleaning.		
42	Regularly clean and disinfect doorknobs, stairs, handrails, and another easy-to-touch equipment.		
43	Maintain open air in indoor places, and continuously monitor change of air condition.		
44	Arrange special anti-epidemic passages and routes for communities.		
45	Those who enter the community are required to wear a mask. Those who refuse to wear a mask are prohibited from entering the community.		
46	Those who enter the community require a body temperature test. Those who refuse to take the temperature test are prohibited from entering the community.		
47	Those who enter the community require disinfection of their hands. Those who refuse are prohibited from entering the community.		
48	Items delivered by home delivery companies and food takeaways are sent to designated storage areas for temporary storage, and residents are notified to collect them by themselves.		
49	Supplies for the quarantined households will be delivered to their door by service staff (gloves required) and the money will be taken (or comply with community regulations).		
50	Set up isolation panels or other items at the service counter, to maintain social distance with residents or visitors.		
51	Carefully monitor people entering and exiting, know their paths and destinations in the community, and remind them not to go to unnecessary places.		
52	When outsiders enter the community, they should fill in the registration form and declaration form as required, and record body temperature for future tracking and data retention.		
53	Special management for brokers who bring client into the community.		
54	Post health education forms and advocate DM or related letters on community bulletin boards.		
55	Announce government guidelines through marquees, electronic bulletin boards, etc. to advocate epidemic prevention.		
56	Residents are invited to study and watch related education materials of the National Health Agency.		
57	The management committee and service staff participate in government’s related epidemic prevention activities.		
58	The management committee and service staff urge residents to avoid participating in crowd gatherings or going to popular attractions.		
59	Advocate keeping social distance.		
60	Take actions to prevent false announcements and negative information.		
61	Change meeting method when holding management committee meetings.		
62	Seek community residents to cooperate and support community epidemic prevention work.		
63	Actively participate in the selection activities of excellent epidemic prevention community organized by the government.		
64	Join government smart community application, government instant messaging, smart community Facebook fan group, etc.		
65	Combine community property management APP to enhance epidemic prevention.		
66	The community creates a Facebook epidemic prevention group to discuss and communicate how to jointly protect home and implement good epidemic prevention management.		
67	Community uses online video to hold meetings.		
68	Establish a community epidemic management information system.		

## Appendix B

**Table A2:** Epidemic Prevention Measures Classification.

12 Types of Epidemic Prevention Measures
**Living assistance measures during the epidemic prevention period**
30. During quarantine, communication and coordination should be enhanced to settle disputes among households.
31. Assist households in home quarantine and independent health management, such as food delivery, ensuring normal operation of water, electricity, gas, and other home supplies.
49. Supplies for the quarantined households will be delivered to their door by service staff (gloves required) and the money will be taken (or comply with community regulations).
**Epidemic prevention propaganda**
21. Establish a communication list of external support units such as hospitals, district chiefs, public institutions, and volunteer groups.
54. Post health education forms and advocate DM or related letters on community bulletin boards.
55. Announce government guidelines through marquees, electronic bulletin boards, etc. to advocate epidemic prevention.
56. Residents are invited to study and watch related education materials of the National Health Agency.
57. The management committee and service staff participate in government’s related epidemic prevention activities.
58. The management committee and service staff urge residents to avoid participating in crowd gatherings or going to popular attractions.
59. Advocate keeping social distance.
60. Take actions to prevent false announcements and negative information.
62. Seek community residents to cooperate and support community epidemic prevention work.
63. Actively participate in the selection activities of excellent epidemic prevention community organized by the government.
**Epidemic prevention distribution**
14. Plan service areas and home delivery storage areas, to reduce chances for residents to contact community service staff.
17. Ask the appointed management company and related third-party vendors propose corresponding management methods during epidemic prevention period in the community.
33. Public areas such as indoor children’s playrooms, multi-functional activity spaces, the elderly’s classrooms, chess rooms, etc. are temporarily closed.
48. Items delivered by home delivery companies and food takeaways are sent to designated storage areas for temporary storage, and residents are notified to collect them by themselves.
**Ordinary epidemic prevention measures**
7. Conduct epidemic prevention exercises at the right time to facilitate the establishment of epidemic prevention concepts and disaster response actions. (Like a fire drill).
8. Record travel history, contact history, and health record.
9. Daily self-health check: test body temperature, put a sticker or badge to show that you are all right.
10. Wear masks, face masks, gloves, and other necessary protective supplies for daily work.
15. Disinfect and clean community service staff’s workplaces daily.
16. Change of air in workplaces. Have windows open as much as possible, and adopt natural change of air requirements.
18. Set emergency plans for suspected infection of community service staff: emergency notification, quarantine, and treatment in accordance with relevant government regulations.
29. Comply with government policies. Carry out necessary control or help with those who refuse home quarantine, in accordance with community regulations.
34. Hand cleaning and disinfection equipment should be placed in all unclosed public facilities for use.
36. The elevator control panel has stickers which is replaced daily. Elevator equipment has cleaning and maintenance records.
37. Keep complete records of environmental disinfection and cleaning (regularly check hand cleaning supplies in public areas, and refill at any time).
39. Cleaning staff work strictly in accordance with specified work standards. For example, you must wear gloves and masks throughout work process, and avoid accidental touch with mouth, nose, and eyes, or splashes of detergent.
42. Regularly clean and disinfect doorknobs, stairs, handrails, and another easy-to-touch equipment.
43. Maintain open air in indoor places, and continuously monitor change of air condition.
44. Arrange special anti-epidemic passages and routes for communities.
**Epidemic prevention teaching and training**
19. Related check forms and control measures are easy to understand, learn, and do.
20. Conduct epidemic prevention and control training for community service staff.
28. Convert complex and difficult community control procedures into easy-to-read and easy-to-understand contents.
**Community facilities and environmental disposal management during the epidemic prevention period**
32. Maintain facilities such as water, electricity, gas, Internet, garbage disposal, air conditioning systems, sewage disposal, toilets, and other facilities in advance to ensure that they are operational.
38. Garbage area is timely cleaned without stacking.
40. Garbage generated during home quarantine shall be collected and processed by professional agencies.
41. Enhance frequency of outdoor public space disinfection, ditch cleaning, tree pruning, water tanks cleaning, and other environmental cleaning.
**Epidemic prevention equipment**
4. Put masks, thermometers, gloves, alcohol, disinfectant, dry cleaning gel, goggles or face masks as needed, and manage epidemic prevention supplies well.
11. Place protective supplies including disposable medical surgical masks, gloves, and isolation plates.
12. Check and monitor the performance of temperature testing equipment such as forehead thermometer and infrared rays.
35. Service counter, entrance, and exit are equipped with cleaning fluid for people entering and exiting.
50. Set up isolation panels or other items at the service counter, to maintain social distance with residents or visitors.
**Epidemic prevention and care**
22. Establish an epidemic prevention care team to actively care and understand the disadvantaged households in the community and surrounding neighborhoods.
23. Provide relevant assistance to economically disadvantaged households whose livelihoods are affected due to the epidemic to stabilize them.
24. During quarantine, domestic violence cases are prone to occur, and households are encouraged to strengthen self-protection. Management committees and community workers enhance attention and care.
25. Recruit short-term care workers in the community to help reduce life difficulties for the elderly, and initiate short-term care workers mechanism.
26. Take the initiative to care for the hearing impaired and visually impaired in the community, and assist in obtaining information so that they can receive the latest information in time.
27. For those who have been listed living alone and have limited mobility, assist government unit in distribution of care masks.
**Community epidemic prevention administrative management**
1. Community independently establishes epidemic prevention checklists.
2. Improve epidemic prevention management rules and standard operating procedures (SOP).
3. Members of the management committee or residents share the work of epidemic prevention, and establish an epidemic prevention response team.
6. Provide medical supplies for front-line property management staff or provide more encouragement and substantial rewards during the epidemic.
61. Change meeting method when holding management committee meetings.
**Smart epidemic prevention systems**
64. Join government smart community application, government instant messaging, smart community Facebook fan group, etc.
65. Combine community property management APP to enhance epidemic prevention.
66. The community creates a Facebook epidemic prevention group to discuss and communicate how to jointly protect home and implement good epidemic prevention management.
67. Community uses online video to hold meetings.
68. Establish a community epidemic management information system.
**Entry restriction**
45. Those who enter the community are required to wear a mask. Those who refuse to wear a mask are prohibited from entering the community.
46. Those who enter the community require a body temperature test. Those who refuse to take the temperature test are prohibited from entering the community.
47. Those who enter the community require disinfection of their hands. Those who refuse are prohibited from entering the community.
**Entry tracking**
51. Carefully monitor people entering and exiting, know their paths and destinations in the community, and remind them not to go to unnecessary places.
52. When outsiders enter the community, they should fill in the registration form and declaration form as required, and record body temperature for future tracking and data retention.
53. Special management for housing agency who bring client into the community.
